# Introduction to the Monte Carlo dose engine COMPASS for BNCT

**DOI:** 10.1038/s41598-023-38648-y

**Published:** 2023-07-24

**Authors:** Wan-Bing Zhong, Jiang Chen, Yi-Chiao Teng, Yuan-Hao Liu

**Affiliations:** 1 Neuboron Therapy System Ltd., Xiamen, Fujian Province People’s Republic of China; 2 National Tsing Hua University, Hsinchu, 30013 Taiwan Republic of China; 3 Nanjing University of Aeronautics and Astronautics, Nanjing, Jiangsu Province People’s Republic of China; 4 Neuboron Medtech Ltd., Nanjing, Jiangsu Province People’s Republic of China

**Keywords:** Particle physics, Techniques and instrumentation, Mathematics and computing, Physics

## Abstract

The Monte Carlo method is the most commonly used dose calculation method in the field of boron neutron capture therapy (BNCT). General-purpose Monte Carlo (MC) code (e.g., MCNP) has been used in most treatment planning systems (TPS) to calculate dose distribution, which takes overmuch time in radiotherapy planning. Based on this, we developed COMPASS (COMpact PArticle Simulation System), an MC engine specifically for BNCT dose calculation. Several optimization algorithms are used in COMPASS to make it faster than general-purpose MC code. The parallel computation of COMPASS is performed by the message passing interface (MPI) library and OpenMP commands, which allows the user to increase computational speed by increasing the computer configurations. The physical dose of each voxel is calculated for developing a treatment plan. Comparison results show that the computed dose distribution of COMPASS is in good agreement with MCNP, and the computational efficiency is better than MCNP. These results validate that COMPASS has better performance than MCNP in BNCT dose calculation.

## Introduction

Boron neutron capture therapy (BNCT) utilizes the properties of boron-containing drugs that have a high capture cross section for thermal neutrons to kill tumor cells using two heavy ions created by the ^10^B(n,α)^7^Li nuclear reaction. The range of these two heavy ions is approximately the size of a cell, so the heavy ions kill only nearby cells and do not damage further cells^1^. When boron-containing drugs selectively accumulate in tumor cells, and cooperated with a suitable neutron source, the effect of local killing of tumor cells while minimizing damage to normal cells can be achieved^2^.

Due to the unique advantages of BNCT in the field of radiation therapy, many BNCT treatment centers are being constructed to treat cancer patients^3–7^. As the core subsystem of precision radiotherapy, the Treatment Planning System (TPS) is applied to calculate the expected dose distribution and determine appropriate irradiation parameters for optimal treatment results. For conventional radiotherapy represented by photon beams, TPS can apply a simplified numerical model-based approach to speed up the calculation. However, this approach is not feasible in BNCT. The BNCT beam contains a mixture of neutrons and photons. The secondary particles of neutrons may have neutrons and photons, which makes it difficult to simplify the dose calculation. In addition, different particles may need different radiobiology weighting factors, which makes it even more complicated to be described in a simplified way. Therefore, the Monte Carlo (MC) calculation is usually applied in radiation simulation in BNCT. Although research on the clinical application of BNCT is comparatively short, several research institutions and researchers have developed TPS applicable to BNCT.

Most TPSs for BNCT treatment use general-purpose MC codes such as MCNP (e.g., NCTPlan^8^, THORPlan^9^) and PHITS (e.g., JCDS^10^, Tsukuba Plan^11^, NeuCure^12^) as their dose calculation engines. Although such a development strategy can reduce the difficulty of TPS development, it is not the best choice, and the reasons are as follows: (1) the general-purpose code is not developed for BNCT and lacks the quality control and testing requirements for medical software, which makes it difficult to ensure its reliability and safety as medical software to meet the regulatory requirements^13^. (2) The general-purpose MC code is powerful and applicable to a wide range of applications and therefore requires many cross-section libraries and subroutines. This, in turn, means that it is difficult to be optimized for specific purposes. A much longer time is required to complete a calculation task, which makes it difficult to handle a large number of patients. When the treatment plan needs to be revised within a short period (e.g., the original irradiation location is found to be unattainable on the day of irradiation), the computational statistical accuracy of the general-purpose MC code will not satisfy the need because the computation time is proportional to the square of the statistical accuracy. (3) The functions of the general-purpose MC code are not fully tailored to BNCT application scenarios, making it difficult to accurately describe BNCT calculation models (especially the beam source definitions).

Very few TPSs for BNCT have their own MC engine, such as SERA (Simulation Environment for Radiotherapy Applications)^14^, but the results of the study show that the dose calculation results from SERA deviate significantly from the dose calculation results of the general-purpose MC code, especially in skins^15^, and therefore it cannot be used as a treatment planning system for precision radiotherapy.

The BNCT dose calculation code employing a GPU-accelerated Monte Carlo (MC) method^16^ significantly reduces computation time. In this study, Lee et al. employ the multi-group Monte Carlo method to calculate neutron flux. The precision of this approach falls short compared to the continuous-energy Monte Carlo method^17^. Thus, most general-purpose Monte Carlo codes prefer the use of the continuous-energy Monte Carlo method due to its superior accuracy. Consequently, Lee et al. emphasize that in the case of a head CT voxel model, the mean absolute percentage errors for neutron flux and absorbed dose are 3.98% and 3.91%, respectively, which is considered unacceptable in radiotherapy^26,27^. This deviation renders the improvement in computation speed insignificant.

For these reasons, we independently developed a dedicated MC engine COMPASS (COMpact PArticle Simulation System) in NeuMANTA (a TPS specifically for BNCT) for BNCT dose calculation and achieved the goal of significantly reducing the computation time without sacrificing the accuracy of dose calculation. In addition, the program customizes the geometric model and source distribution definitions for BNCT specifically and makes the calculation model closer to the real application scenarios of BNCT. Thus, the dose distribution of patients can be calculated more efficiently and accurately.

## Methods

### Introduction of NeuMANTA

NeuMANTA is designed with specific features tailored for BNCT treatment planning, including a user-friendly visual interface, a medical image processing module, a voxel model building module, a database for relative biological effectiveness (RBE) and compound biological effectiveness (CBE), a dose calculation engine, and a report generation module. NeuMANTA supports the DICOM format, which is widely adopted in the medical field, including support for DICOM-RT.

In creating a treatment plan, NeuMANTA first reads the patient image data and automatically generates multiple human tissue materials based on the Hounsfield Unit (HU) value of the CT image data. Within NeuMANTA, users can select the location and direction of the radiation source and the type of collimator to be used. After setting the treatment parameters, an input file for COMPASS dose calculation is generated and submitted to COMPASS for calculation. Upon completion, a physical dose output file is generated, which NeuMANTA can read and use to calculate the equivalent biological dose for each voxel based on the CBE and RBE of various materials. The user then determines the treatment plan and whether to accept the irradiation condition based on the biological dose distribution.

### Workflow of COMPASS

**Figure 1:**
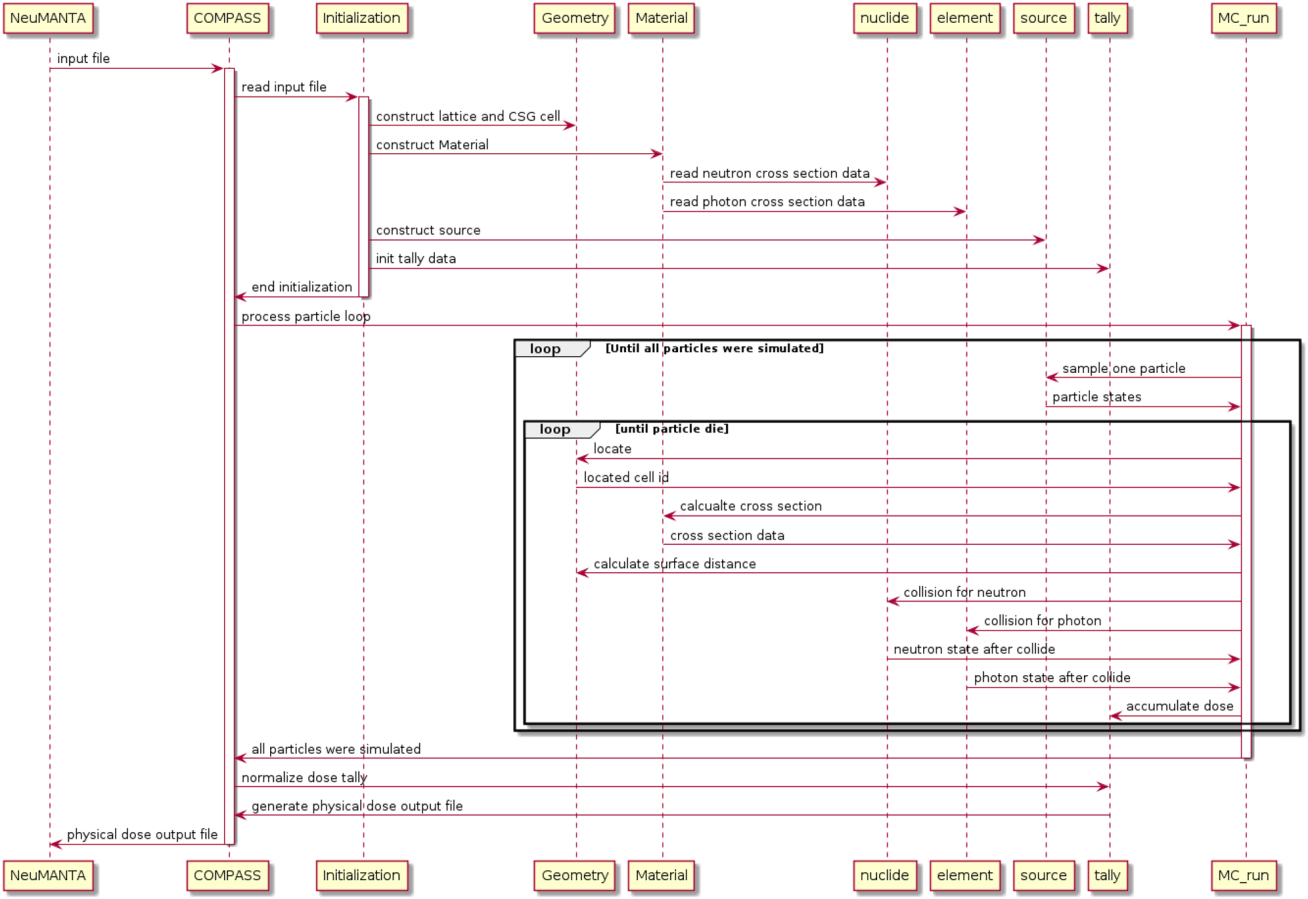
Workflow of COMPASS.

Considering the lengthy computation time of general-purpose MC codes and the challenges in accurately describing the BNCT calculation model using numerical methods, we developed our own MC calculation code, COMPASS. Figure 1 illustrates the timing chart for various modules of COMPASS involved in the formulation of a radiotherapy plan.

First, NeuMANTA generates the input file required for COMPASS calculation based on the parameters provided by the user. COMPASS reads the input file and begins the initialization process, which includes creating geometry, creating materials, reading neutron cross-section databases for each nuclide, and reading photon cross-section databases for each element. The source is created based on the input file information. Memory is allocated for the tally vector according to the grid size, and the vector is initialized to zero.

Next, the “MC_run” module in COMPASS simulates each particle's history. This involves sampling particle states, locating particle positions, calculating cross-sections of particle interactions with materials, calculating distances to the nearest surface, processing particle collisions with materials, and accumulating doses deposited in voxels. This process is repeated until all particles have been simulated.

Finally, tally data is normalized by the total number of particles, and a physical dose output file is generated for NeuMANTA.

### Dose calculation of COMPASS

In the COMPASS system, the interactions of neutrons and photons with matter are meticulously treated to mirror the procedures incorporated in the general-purpose Monte Carlo (MC) code, thereby bolstering the validity of the dose calculation results^17^. For photons, this involves simulations of phenomena such as the photoelectric effect, Compton scattering, pair production, and more. Similarly, for neutrons, the handling includes processes like elastic scattering, inelastic scattering, capture reactions, and thermal scattering, among others. This comprehensive approach, encompassing a wide array of interactions, ensures that COMPASS's treatment aligns with the methodology used in the general-purpose MC code, thereby validating the accuracy of the calculated dose outcomes. The charged particle was deposited locally in COMPASS since the dose in BNCT is mainly contributed by heavy ions rather than electrons. The dose estimation in COMPASS is calculated with the track length estimation, which is calculated by Eq. 1:

1 $$D\left( E \right) = \phi \left( E \right) \times \mathop \sum \limits_{i} \left( {\rho_{i} \sigma_{i} \left( E \right)H_{i} \left( E \right)} \right)$$

where $\phi \left( {\text{E}} \right)$ is the flux of the particle in each voxel and is determined by the motion history of the particle; i corresponds to the ith nuclide of the material at that voxel, $\rho_{i}$ is the density of the ith nuclide, ${\upsigma }_{{\text{i}}} \left( {\text{E}} \right)$ is the total reaction cross-section of the ith nuclide with particle energy E, and ${\text{H}}_{{\text{i}}} \left( {\text{E}} \right)$ is the average energy deposition of the collision between the particle with energy E and the ith nuclide. Both the reaction cross section and the average energy deposition are calculated by the nuclear data library based on energy interpolation. If the particle type is a photon, photon dose was accumulated by Eq. 1. Neutron dose was accumulated in the same way. If the material was boron contained, only the neutron dose contributed by ^10^B atoms was added to the boron dose.

To reduce the dose calculation time, several optimization methods were used in COMPASS.

All of the general-purposed MC algorithms explained in the next sections were mainly based on MCNP’s reference^17^. MCNP is a general-purpose MC N-Particle code; now in the version MCNP6^18^, developed at Los Alamos National Laboratories, it is one of the most commonly used general-purpose MC codes. Since several TPSs for BNCT use MCNP as their dose calculation engine, and the dose calculation output file of MCNP6 can also be read by NeuMANTA, so we mainly use MCNP6 as the comparison software for COMPASS.

### Energy interval search algorithm

Cross-section calculation is one of the most time-consuming steps in simulating particle history^19^. The cross-section data of several energy nodes were provided in the database, and the energy of the particle is often in the middle of two adjacent energy nodes. Therefore, the key step of the cross-section calculation is to find the energy interval of the database corresponding to the current particle energy.

**Figure 2:**
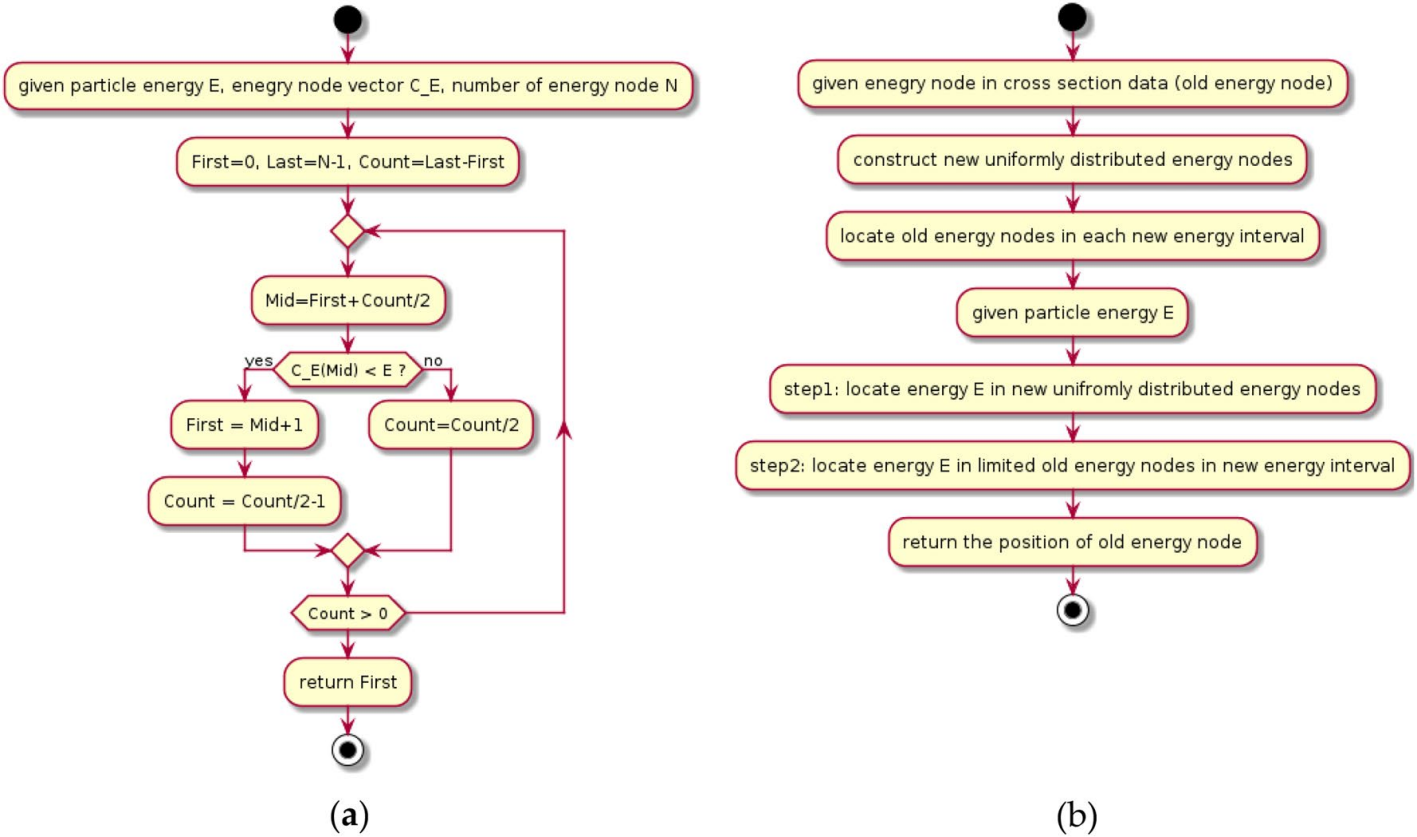
Workflow of binary search (**a**) and hash search algorithm (**b**).

Given that the energy nodes of cross-section data are not uniformly distributed, binary search is frequently employed for energy interval searches, as depicted in Fig. 2a. The fundamental principle of binary search is to ascertain whether the particle energy resides in the upper or lower half of the energy nodes during each iteration. The search range is then narrowed based on the determination, and the process is repeated until the final node position is identified. The relationship between the binary search time and the total number of energy nodes is logarithmic. The more energy nodes there are, the longer the binary search time. The number of energy nodes in the cross-section database was usually as high as thousands and even tens of thousands; therefore, the calculation time of the neutron cross-section processing is costly.

To reduce the time of the cross-section calculation, a hash search was used to replace the binary search in COMPASS. The workflow of the hash search algorithm^16^ is shown in Fig. 2b.

In contrast, the hash search algorithm necessitates an additional preparatory step compared to the binary search, as illustrated in Fig. 2b. This process commences with the establishment of uniformly distributed energy nodes using logarithmic interpolation. The maximum (Emax) and minimum (Emin) values of the new energy nodes depend on the range of the old energy nodes in the cross-section database. The scope of the new energy node must encompass that of the old energy node. Subsequently, the position of each old energy node at the new energy node is calculated. This portion of the work can be completed during the initialization step when reading the cross-section data library, thereby not impacting the computational speed of particle simulation.

For a given particle energy E, the new interval grid is calculated first using Eq. 2,

2 $$u\_grid = \left( {\ln E - \ln E_{min} } \right)*M/\left( {\ln E_{max} - \ln E_{min} } \right),$$

where M represents the total number of new energy nodes. If M is sufficiently large, the number of old energy nodes in a given new energy interval becomes very limited (e.g., one), allowing the old energy node's location to be quickly identified. As a result, the hash search time is no longer correlated with the number of old energy nodes and is notably faster than the binary search. The primary drawback of the hash search method is its increased memory usage for storing new energy nodes. However, in boron neutron capture therapy (BNCT) dose calculations, the memory required for the cross-sectional database itself is relatively small, rendering this disadvantage acceptable. This contrasts with nuclear reactor calculations, where the energy nodes of fission nuclides are extensive, numbering in the hundreds of thousands, causing the cross-section database to occupy a significant amount of memory.

### Transportation optimization method

**Figure 3:**
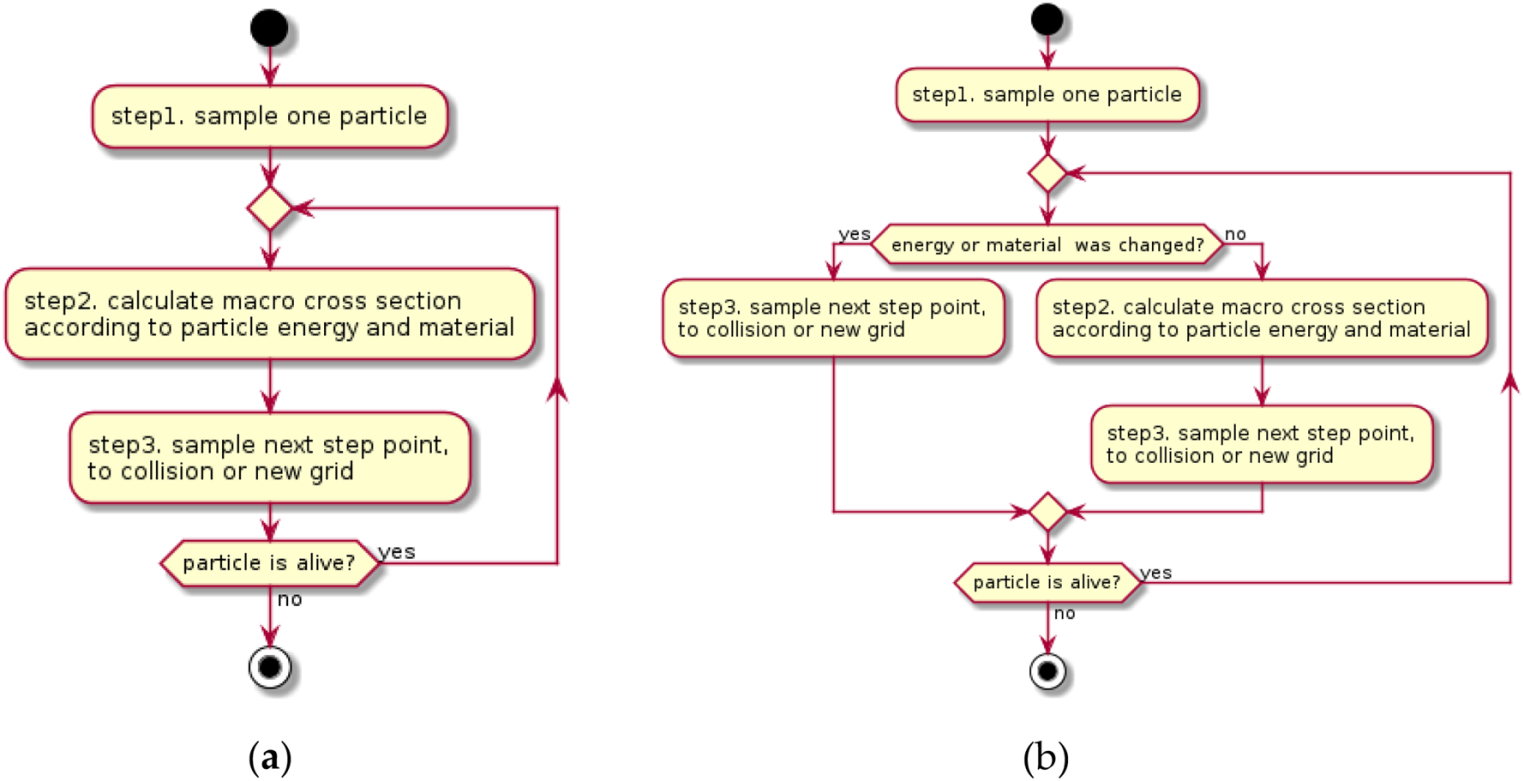
Workflow of simulating particle history by (**a**) general-purpose code and (**b**) COMPASS.

The main geometric model in the BNCT dose calculation is a lattice. In BNCT, the lattice is a uniformly structured grid, and each grid was filled with one material. To make the model closer to the real situation, the grid was finely divided and caused a high probability that the adjacent voxel was filled with the same material. The workflow of simulating one particle in the general-purpose MC code (e.g., MCNP) is shown in Fig. 3a.

In Step 2, if particles are transported from one voxel to the adjacent voxel, the macro cross-section will be calculated in the new voxel. In this step, when the voxel material and particle energy do not change (since particle energy could be changed only in a collision event), the macro cross-section of the new voxel is the same as before, and it is not necessary to calculate the macro cross-section twice. In COMPASS, if particles are transported to other voxels (energy was not changed) where the material is the same as before, Step 2 will be skipped and go to Step 3 (shown in Fig. 3b, which will reduce the time of calculating the cross-section.

### Dose tally optimization method

In any Monte Carlo (MC) codes, both the mean value and statistical error are calculated, as long as the MC algorithm is utilized. The mean value corresponds to the dose required, while the statistical error serves as an indicator for determining whether convergence is achieved. Consequently, it is crucial to accurately calculate the mean value, while the statistical error does not need to be as precise. The mean value was calculated by Eq. 3.

3 $$mean = \frac{{\mathop \sum \nolimits_{i = 0}^{N} t_{i} }}{N}$$

The statistical error was calculated by Eq. 4.

4 $$error = \sqrt {\frac{{\left( {\mathop \sum \nolimits_{i = 0}^{N} t_{i}^{2} } \right)/N - mean^{2} }}{N - 1}}$$

where t_i_ is the dose tally of each particle, and N is the total number of particles. From Eqs. 3 and 4, it is enough to calculate the mean and error by accumulating t_i_ and t_i_^2^.

When particles pass through the grid, they leave tracks, and the dose contribution of these tracks were calculated by Eq. 1. If a particle passes through the same voxel twice, and the dose contribution was t_1_ and t_2_, the total dose contribution t_i_ = t_1_ + t_2_, and the total error contributes t_i_^2^ = (t_1_ + t_2_)^2^. However, determining whether a particle has ever passed through a given grid is a time-consuming step because there are too many voxels. In COMPASS, we skip this step and accumulate the tally and error as if the particle enters two different voxels, so the total dose contributes t_i_ = t_1_ + t_2_, and total error contribute t_i_^2^ = t_1_^2^ + t_2_^2^. The mean value is the same as before, but since t_1_^2^ + t_2_^2^ < (t_1_ + t_2_)^2^, the error will be underestimated. If the error is not quite a deviation from the real value, and the calculation time is significantly reduced, it is worth using this time-saving method listed above.

We performed calculations on neutron doses for various tissues in a model to confirm the efficiency of the implemented algorithms. Table 1 displays the dose calculation results and statistical errors of each tissue before and after using the dose tally optimization algorithm. The optimization algorithm demonstrates no impact on the dose results, and the statistical error calculated after optimization is 10% to 20% lower than the true statistical error.

**Table 1:** Neutron dose and average voxel error before and after optimization.

Tissue	Neutron dose before optimization (MeV/g)	Average voxel neutron dose error before optimization (%)	Neutron dose after optimization (MeV/g)	Average voxel neutron dose error after optimization (%)
Brain	2.69581E−8	9.24	2.69581E−8	7.91
Brain stem	2.89934E−8	6.61	2.89934E−8	5.58
Spinal cord	2.61014E−8	7.34	2.61014E−8	6.27
Mandible	5.40092E−8	8.42	5.40092E−8	7.44
Mucosa	5.49545E−8	7.38	5.49545E−8	6.30
Tumour	2.04912E−7	5.34	2.04912E−7	4.56

Next, we introduce the current functions of COMPASS.

### Introduction of COMPASS functions

The cross-section library applied in COMPASS is of the Hierarchical Data Format, Version 5 (HDF5) format^20^ (version ENDF/B-VIII.0^21^, which is firstly used in OpenMC code^22^). Most general-purpose MC codes (e.g., MCNP and PHITS) are of the ACE^23^ (A Compact ENDF) format. HDF5 is specifically applied to large-scale data input and output, which is much more efficient than the ACE format, so COMPASS is also faster in loading cross-section data.

All functions required for BNCT dose calculation are available in COMPASS, such as source sampling, physical geometry construction, and dose tally. To reduce the calculation time, the MPI library^24^ and OpenMP^25^ instructions were used, which increased the computing speed. Users can reduce the computing time to a few minutes as long as the number of computer cores is sufficient.

An accurate definition of radiation sources is essential to ensure the correctness of the dose calculation results. COMPASS has a flexible source definition function that allows the definition of various types of radiation sources. Since both neutrons and photons are included in the radiation sources of BNCT, COMPASS allows users to define different types of particle sources and different types of distributions. In addition, COMPASS adds the spatial rotationally symmetrical distribution type of radioactive sources, which is the type that best matches the real BNCT radioactive source distribution.

Both lattice and constructive solid geometry (CSG) definitions are allowed in COMPASS. However, since the grid model used for BNCT dose calculation is relatively simple, with each voxel containing only one material, COMPASS directly defines voxel meshes in the form of structural meshes, simplifying the logic of geometric definition and processing. COMPASS does not implement multi-layer geometric definition functions, which limits the ability to make direct comparisons. Nevertheless, this optimization contributes to the overall computing efficiency.

## Results

### Algorithm acceleration effect of the optimization algorithms described before

To test the acceleration effect of different algorithms, describe before, we implemented both the algorithms of the general-purpose MC code and the new optimized algorithm in COMPASS and compared the difference in their computational speed. Table 2 gives the acceleration effects of the three optimization algorithms, and the acceleration effect for total calculation time is calculated by Eq. 5:

5 $$accelerate\;effect = \frac{{T_{before} - T_{after} }}{{T_{before} }} \times 100\%$$

**Table 2:** Acceleration effect of the three algorithms.

Method Before Optimization	Method After Optimization	Accelerate effect (%)
Energy binary search	Energy hash search	10
Transport method of MCNP	Transport method of COMPASS	12
Dose tally method of MCNP	Dose tally method of COMPASS	30

In Table 2, both the first and second optimization algorithms reduce the computation time of macro cross sections, and the third optimization algorithm reduces the time of dose tally. The first two optimization algorithms have no side effects at all, but the third optimization algorithm underestimates the statistical error of the dose.

The test results show that the third optimization algorithm leads to an approximately 10% to 20% underestimation of the statistical error (data was given in Table 1 the statistical error is the same as not using the third optimization algorithm). Since the statistical error is only used for the assessment of whether the calculation results converge, this cost is worthwhile since the significant improved (30%) in calculation speed while the true statistical error was not changed.

### Accuracy verification of dose between COMPASS and MCNP6

**Figure 4:**
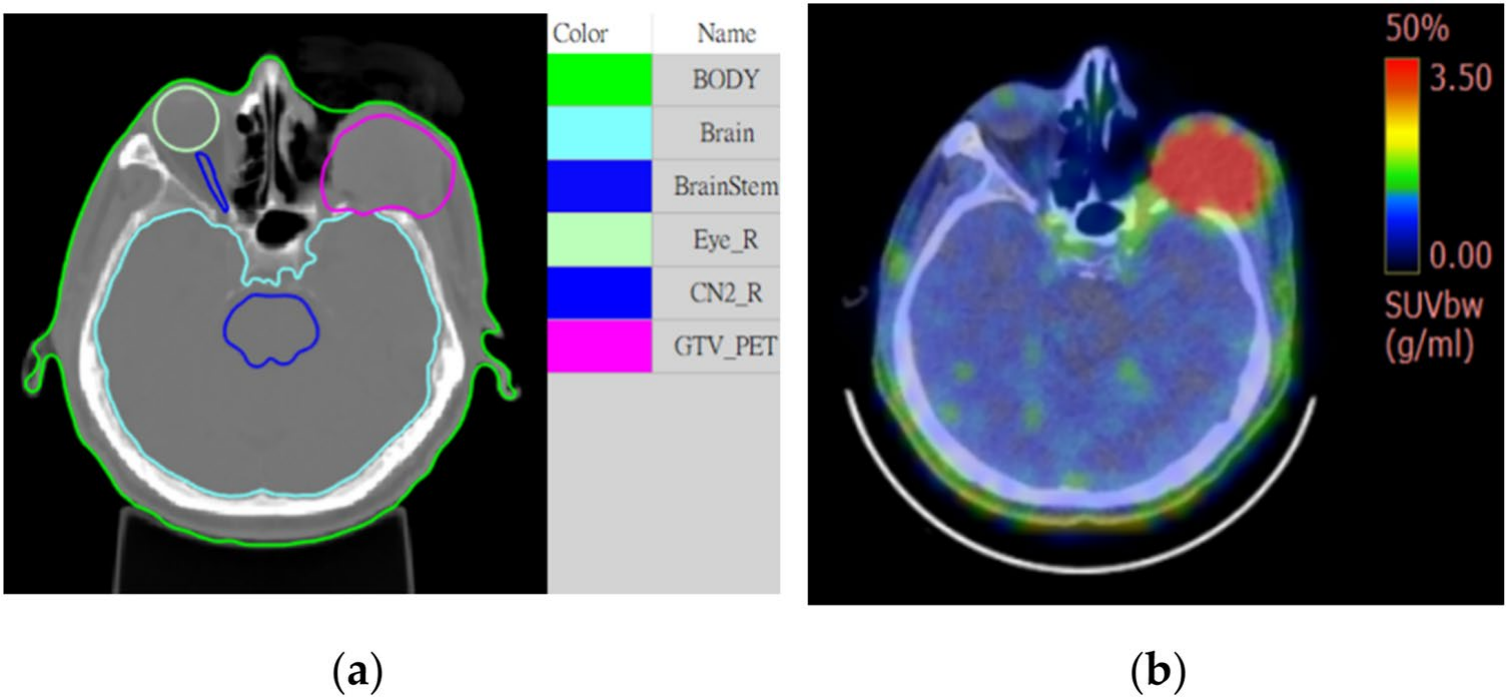
(**a**) Shows the computer tomography (CT) image with ROI information retrieved from the RTSTRUCT file, where the tag Brain represents the normal brain, and the tag GTV_PET represents the tumor region, which is defined based on the PET image. (**b**) F-BPA PET/CT fusion image.

Several actual head and neck cancer cases have been compared using COMPASS and MCNP6, and one of them will be presented in this section. The head and neck cancer model is shown in Fig. 4.

In this model, the blood boron concentration is 25 ppm, and the tumor boron concentration is 75 ppm. The voxel size measures 0.75 mm × 0.75 mm × 5 mm, and the number of grids is 259 × 289 × 34. The material of each voxel is automatically transformed by the HU value in NeuMANTA. The geometry and material composition are consistent in COMPASS and MCNP6. The treatment source's irradiation direction is from right to left. The proportions of thermal neutrons (< 1 eV), epithermal neutrons (1 eV-0.1 MeV), and fast neutrons (> 0.1 MeV) are 1%, 98%, and 1%, respectively.

**Figure 5:**
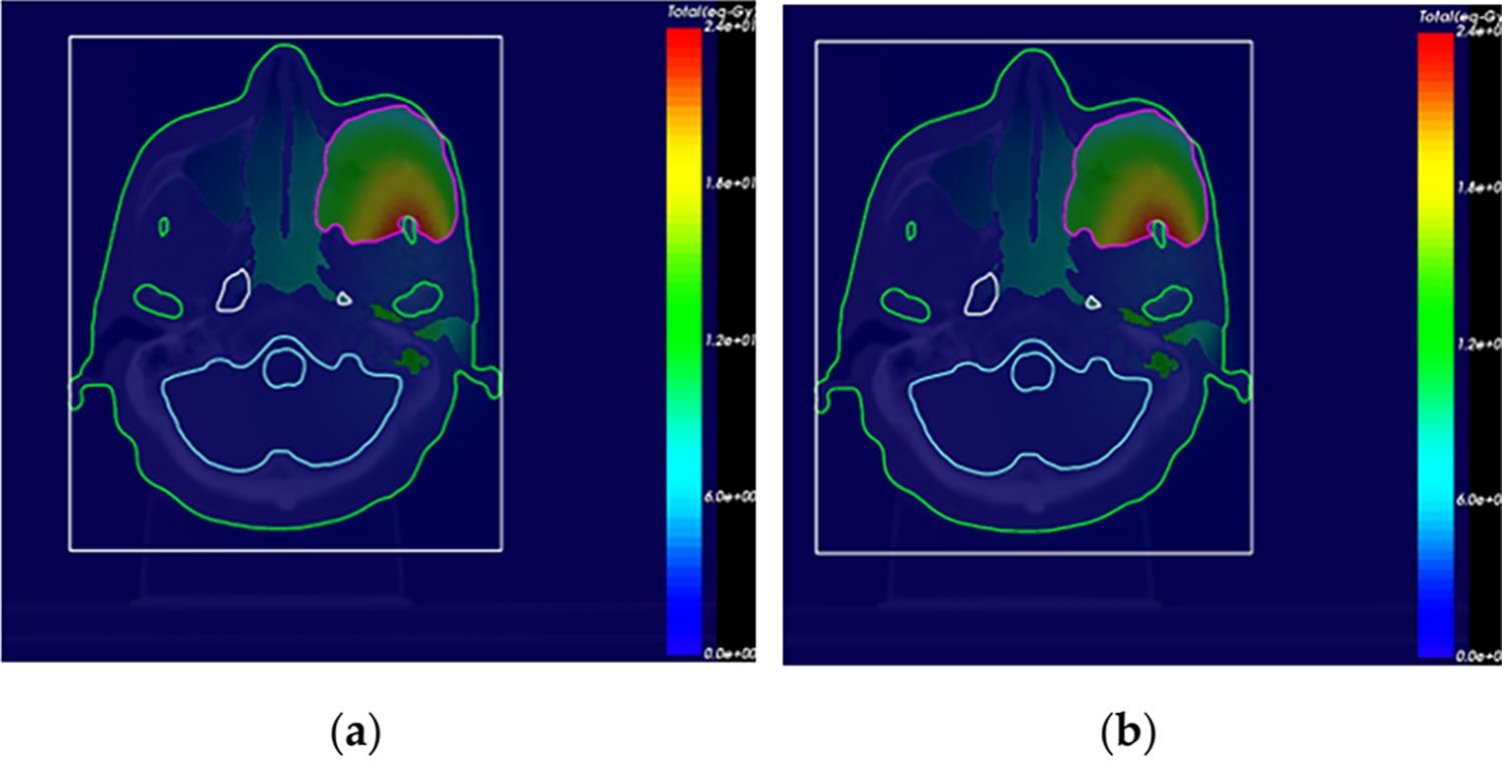
Dose distribution calculated by (**a**) COMPASS and (**b**) MCNP6.

A total number of 2 × 10^8^ neutrons were simulated by COMPASS and MCNP6, and the boron dose, neutron dose, and photon dose were calculated for each voxel. The distribution of the neutron dose at a given CT slice calculated by COMPASS and MCNP is given in Fig. 5.

The results of the neutron dose distribution calculated by COMPASS and MCNP can be seen visually in Fig. 5, and it is nearly impossible to tell the difference by eye. Nonetheless, the calculation results of the two codes must be compared quantitatively.

The mean and maximum doses are important considerations in treatment plan-making. Tables 3, 4, and 5 show the relative deviations of the mean and maximum doses in tumors as well as normal tissues calculated by COMPASS and MCNP (see Eq. 6 for calculation method of relative deviation). For the tumor ROI, the minimum dose is of interest because the cold spot may lead to recurrence in the future. The tumor minimum dose component results are listed in Table 6. Relative statistical errors of dose were given in parentheses following the dose data in Tables 3, 4, 5, and 6.

6 $$relative\;deviation = \frac{{\left| {D_{MCNP} - D_{COMPASS} } \right|}}{{D_{MCNP} }} \times 100\%$$

**Table 3:** The mean and maximum boron doses of each tissue were calculated using COMPASS and MCNP.

Tissue	Mean Boron Dose (MeV/g) COMPASS	Mean Boron Dose (MeV/g) MCNP6	Deviation of Mean Dose (%)	Max. Boron Dose (MeV/g) COMPASS	Max. Boron Dose (MeV/g) MCNP6	Deviation of Max. Dose (%)
Brain	2.40562e−08 (0.04%)	2.40724e−08 (0.01%)	0.07	8.16418e−08 (0.7%)	8.20211e−08 (0.88%)	0.46
Brain Stem	2.66256e−08 (0.12%)	2.65779e−08 (0.05%)	0.18	4.36625e−08 (1.0%)	4.4742e−08 (1.15%)	2.41
Spinal Cord	2.36025e−08 (0.18%)	2.3652e−08 (0.1%)	0.21	3.42794e−08 (1.1%)	3.43419e−08 (1.28%)	0.18
Mandible	4.58901e−08 (0.06%)	4.59036e−08 (0.04%)	0.03	1.29109e−07 (0.8%)	1.29302e−07 (0.89%)	0.15
Mucosa	4.97985e−08 (0.07%)	4.98503e−08 (0.03%)	0.10	1.39289e−07 (0.8%)	1.39973e−07 (0.91%)	0.49
Tumor	1.95008e−07 (0.05%)	1.95342e−07 (0.02%)	0.17	3.17612e−07 (0.8%)	3.17639e−07 (0.93%)	0.01

**Table 4:** The mean and maximum neutron doses of each tissue were calculated using COMPASS and MCNP.

Tissue	Mean Neutron Dose (MeV/g) COMPASS	Mean Neutron Dose (MeV/g) MCNP6	Deviation of Mean Dose (%)	Max. Neutron Dose (MeV/g) COMPASS	Max. Neutron Dose (MeV/g) MCNP6	Deviation of Max. Dose (%)
Brain	2.69623e−08 (0.04%)	2.6901e−08 (0.01%)	0.23	9.149e−08 (0.8%)	9.15134e−08 (0.88%)	0.03
Brain Stem	2.89576e−08 (0.12%)	2.88177e−08 (0.05%)	0.48	4.74997e−08 (1.0%)	4.85396e−08 (1.15%)	2.14
Spinal Cord	2.61149e−08 (0.17%)	2.60848e−08 (0.09%)	0.12	3.72768e−08 (1.12%)	3.73032e−08 (1.29%)	0.07
Mandible	5.42908e−08 (0.06%)	5.42403e−08 (0.03%)	0.09	1.50867e−07 (0.78%)	1.50684e−07 (0.88%)	0.12
Mucosa	5.49567e−08 (0.07%)	5.49381e−08 (0.03%)	0.03	1.52002e−07 (0.82%)	1.52226e−07 (0.91%)	0.15
Tumor	2.05052e−07 (0.05%)	2.05235e−07 (0.02%)	0.09	3.29746e−07 (0.79%)	3.28645e−07 (0.82%)	0.34

**Table 5:** The mean and maximum photon doses of each tissue were calculated using COMPASS and MCNP.

Tissue	Mean Photon Dose (MeV/g) COMPASS	Mean Photon Dose (MeV/g) MCNP6	Deviation of Mean Dose (%)	Max. Photon Dose (MeV/g) COMPASS	Max. Photon Dose (MeV/g) MCNP6	Deviation of Max. Dose (%)
Brain	1.9044e−08 (0.06%)	1.89059e−08 (0.01%)	0.73	4.63768e−08 (2.6%)	4.70544e−08 (2.64%)	1.44
Brain Stem	2.54461e−08 (0.17%)	2.53584e−08 (0.09%)	0.34	3.67831e−08 (2.8%)	3.58876e−08 (3.02%)	2.50
Spinal Cord	1.90341e−08 (0.29%)	1.89906e−08 (0.18%)	0.23	2.65069e−08 (3.6%)	2.7334e−08 (3.4%)	3.03
Mandible	2.65201e−08 (0.11%)	2.62628e−08 (0.05%)	0.97	6.38508e−08 (2.7%)	6.35785e−08 (2.88%)	0.43
Mucosa	1.93186e−08 (0.11%)	1.91785e−08 (0.04%)	0.73	4.07593e−08 (2.7%)	4.04472e−08 (2.72%)	0.77
Tumor	2.70869e−08 (0.08%)	2.67898e−08 (0.03%)	1.10	4.10249e−08 (2.6%)	4.09453e−08 (2.62%)	0.19

**Table 6:** The minimum tumor dose was calculated using COMPASS and MCNP.

Dose component	Tumor Min. Dose (MeV/g) COMPASS	Tumor Min. Dose (MeV/g) MCNP6	Deviation of Min. Dose (%)
Boron dose	3.7578e−08 (2.04%)	3.72227e−08 (2.36%)	0.95
Neutron dose (Include boron dose)	5.71564e−08 (1.63%)	5.75069e−08 (1.73%)	0.61
Photon dose	1.2466e−08 (4.47%)	1.24182e−08 (4.76%)	0.38

From Tables 3, 4, 5, and 6, the maximum relative deviation of the max. boron and neutron doses calculated by COMPASS and MCNP are 2.14% and 2.41% found in the brain stem; the deviation may be decreased if the statistical errors could be further decreased. The relative deviations for mean boron and neutron doses are smaller than 0.5%. The relative deviations in the photon dose results are larger because the statistical errors are larger. The maximum relative deviation of the mean photon dose is 1.1% in the tumor, and the maximum relative deviation of the maximum photon dose is 3.03% in the spinal cord. A difference of 3% is tolerable in treatment plan-making^26,27^. Note that although the difference in photon dose is larger, its contribution to the total dose is small. In addition, the larger deviations may be improved by further decreasing their statistical errors. According to the calculated results, we can conclude that there is no significant difference in the calculations done by COMPASS and MCNP – the results generated by both codes are equally accurate.

**Figure 6:**
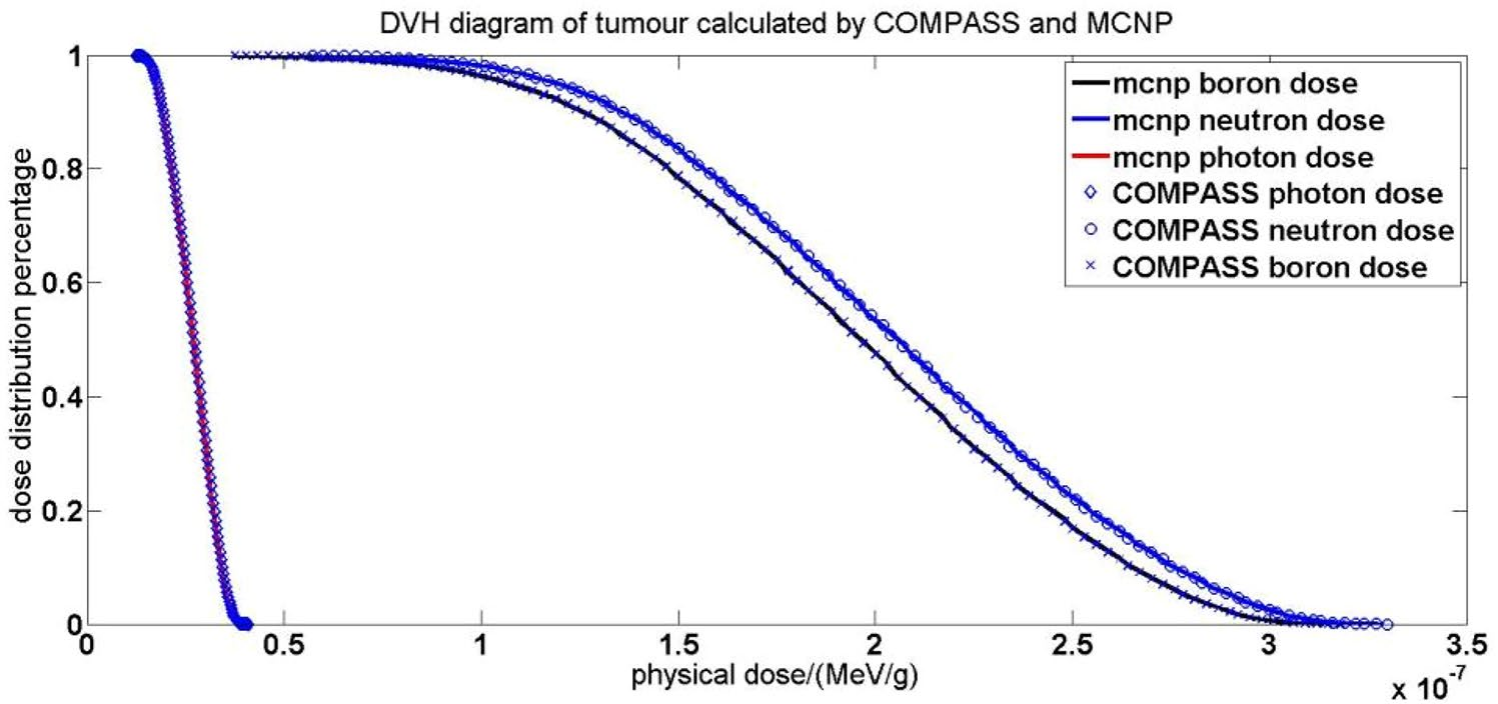
DVH diagram of MCNP6 and COMPASS.

Another data point of interest to the user is the DVH (dose-volume histogram), by which the user can clearly see what percentage of the tissue volume is within the safe dose range. The DVH as per dose calculations of COMPASS and MCNP are given in Fig. 6.

The results in Fig. 6 show that the DVH of COMPASS and that of MCNP almost exactly overlap for all three dose components, proving the consistency of the calculated results of both programs.

In order to succinctly illustrate the precision of COMPASS within diverse head and neck cancer or brain cancer models, the parameter of gamma pass rate was employed. This metric delineates the level of congruity between the dosimetric outcomes computed by COMPASS and MCNP6. Originally implemented in gamma radiotherapy, this method lends itself well to an extension to BNCT.

The gamma pass rate is determined by the ratio of voxels where the gamma function^28^ is less than 1. The gamma function of an individual voxel was calculated using Eq. 7:

7 $$\gamma \left( {\overset{\lower0.5em\hbox{$\smash{\scriptscriptstyle\rightharpoonup}$}}{{r_{r} }} } \right) = \min \left\{ {\sqrt {\frac{{r^{2} \left( {\overset{\lower0.5em\hbox{$\smash{\scriptscriptstyle\rightharpoonup}$}}{{r_{e} }} , \overset{\lower0.5em\hbox{$\smash{\scriptscriptstyle\rightharpoonup}$}}{{r_{r} }} } \right)}}{{\Delta d^{2} }} + \frac{{\delta^{2} \left( {\overset{\lower0.5em\hbox{$\smash{\scriptscriptstyle\rightharpoonup}$}}{{r_{e} }} , \overset{\lower0.5em\hbox{$\smash{\scriptscriptstyle\rightharpoonup}$}}{{r_{r} }} } \right)}}{{\Delta D^{2} }}} } \right\}\forall \left\{ {\overset{\lower0.5em\hbox{$\smash{\scriptscriptstyle\rightharpoonup}$}}{{r_{e} }} } \right\}$$

Here $$r\left( {\overset{\lower0.5em\hbox{$\smash{\scriptscriptstyle\rightharpoonup}$}}{{r_{e} }} , \overset{\lower0.5em\hbox{$\smash{\scriptscriptstyle\rightharpoonup}$}}{{r_{r} }} } \right)$$ represents the spatial distance between the evaluated dose point and the reference dose point, while $$\delta \left( {\overset{\lower0.5em\hbox{$\smash{\scriptscriptstyle\rightharpoonup}$}}{{r_{e} }} , \overset{\lower0.5em\hbox{$\smash{\scriptscriptstyle\rightharpoonup}$}}{{r_{r} }} } \right)$$ denotes the variance between the evaluated dose at position $$\overset{\lower0.5em\hbox{$\smash{\scriptscriptstyle\rightharpoonup}$}}{{r_{e} }}$$ and the reference dose at position $$\overset{\lower0.5em\hbox{$\smash{\scriptscriptstyle\rightharpoonup}$}}{{r_{r} }}$$. $$\Delta$$ d serves as the distance-to-agreement criterion, while $$\Delta$$ D acts as the dose difference criterion.

For these comparative analyses, the dose difference criterion was established at 1% of the maximal dose (in Tables 3, 4, and 5, it can be seen that the average dose of normal tissue is much greater than 1% of the maximum dose of the tumor) and the distance-to-agreement criterion was designated as 1 mm. Only voxels with doses exceeding 1% of the maximum dose were included in the comparison, as regions with low doses are clinically insignificant in terms of treatment impact. A diminution of the dose difference criterion and the distance-to-agreement criterion corresponds with an elevation in the gamma pass rate (with a maximum value of 1), indicating enhanced consistency between the two dose distributions.

In these comparative studies, the gamma pass rates for dose distribution, as computed by COMPASS and MCNP6 for five head and neck or brain cancer models, were recorded as 99.78%, 98.79%, 98.31%, 99.32%, and 99.51% respectively. These values, being near 1, affirm the consistency between the dose calculation outcomes of COMPASS and MCNP6 across various models.

### Computational speed comparison between COMPASS and MCNP

After verifying the accuracy of the dose calculation results of COMPASS, the computing efficiency of COMPASS is tested. The computing efficiency of the MC code is characterized by the FOM (Figure of Merit). The FOM is defined by the following equation:

8 $$FOM = {\raise0.7ex\hbox{$1$} \!\mathord{\left/ {\vphantom {1 {R^{2} T}}}\right.\kern-0pt} \!\lower0.7ex\hbox{${R^{2} T}$}}$$

where R is the relative standard deviation and T is the computation time. For a given MC code, the computation time is usually inversely proportional to the square of the relative standard deviation, i.e., to decrease the relative standard deviation by half, the computation time must be increased four times. Therefore, a larger FOM indicates higher computing efficiency.

To avoid the effect of parallel efficiency on computing efficiency, the computing efficiency is compared based on a single central processing unit (CPU) core. For this model, the single core computation time is 22,243 s for COMPASS and 57,491 s for MCNP6. Table 7 shows the mean statistical error of the three type doses in tumors calculated by the two programs and the comparison of FOM results. (The statistic error of COMPASS was calculated without dose tally optimization method, so the statistic error was not underestimated).

**Table 7:** Comparison of FOM for COMPASS and MCNP.

Dose	MCNP6 result error	MCNP6 FOM value (s^−1^)	COMPASS result error	COMPASS FOM value (s^−1^)	FOM ratio (COMPASS/MCNP6)
Boron dose	0.0122	0.117	0.0123	0.297	2.538
Neutron dose	0.0119	0.123	0.0120	0.312	2.622
Photon dose	0.0324	0.0166	0.0316	0.045	2.711

The results in Table 7 show that the FOM of COMPASS is larger than that of MCNP6, approximately 2–3 times larger than the FOM of MCNP6, implying that the computing efficiency of COMPASS is 2–3 times faster than the computing efficiency of MCNP6.

Table 7 demonstrates a significantly greater acceleration effect compared to Table 2 (2–3 times compared to 50%). This is because Table 2 compares the calculation speed of COMPASS using different algorithms, whereas Table 7 compares COMPASS and MCNP results. Since COMPASS and MCNP are independent software, different code implementations may also impact performance. MCNP's complex functionality can also result in more intricate computational logic, potentially causing a loss of some performance. This is why the performance improvement shown in Table 7 can reach 2–3 times.

In addition, COMPASS supports parallel computing. The calculation time with a single core is over 20,000 s, which is too long. A multi-core computer can significantly reduce the computation time of COMPASS. The test results for the parallel computing of COMPASS show that on a 14-core Intel® Core™ i9-7940X X-series Processor, the computation speed of COMPASS can be increased to 13.96 times of a single core, and the computation time is reduced to less than 30 min, while the calculation time of MCNP6 is more than one hours with the same calculation condition as COMPASS. The dose computation speed is in line with the treatment needs.

## Discussion and conclusion

Owing to the slow dose computation speed of general-purpose MC codes such as MCNP6 and the challenges associated with meeting medical software requirements, we developed our Monte Carlo dose engine, COMPASS. COMPASS demonstrates comparable dose computation accuracy to MCNP6, while its computation time is only 30–50% of MCNP6's computation time. However, since COMPASS is specifically designed for BNCT, it only features two functions: dose calculation and reaction rate calculation. Additionally, as there is no fission in the BNCT treatment scenario, COMPASS does not include the code for fission physical processes. Moreover, due to the simplified geometric definition, defining complex geometry in COMPASS is more challenging than in MCNP.

Besides the optimization algorithms mentioned above, other variance reduction techniques can be employed in BNCT dose calculation, such as weight windows and spatial importance. We are also contemplating the introduction of GPU acceleration for BNCT dose calculations, while ensuring the accuracy of these calculations remains uncompromised.

In summary, COMPASS has a significant advantage over MCNP6 in dose calculation. Test results reveal that COMPASS's computation time can be further reduced to a reasonable range on a high-performance CPU. Currently, the calculation of a real head-and-neck tumor case can be completed within 10 min using four 14-core Intel® Core™ i9-7940X X-series processors running in parallel. We are confident that COMPASS could complete a case within 5 min in the near future, due to both improvements in calculation efficiency and advancements in CPU computation power. COMPASS enables the use of more complex voxel models and dose modeling for BNCT.

## Data Availability

The datasets used and/or analyzed during the current study are available from the corresponding author on reasonable request.
